# MicroRNA-199a-5p promotes tumour growth by dual-targeting PIAS3 and p27 in human osteosarcoma

**DOI:** 10.1038/srep41456

**Published:** 2017-01-25

**Authors:** Chen Wang, Ximing Ba, Yu Guo, Defang Sun, Haoyang Jiang, Wentao Li, Zhen Huang, Guangxin Zhou, Sujia Wu, Junfeng Zhang, Jiangning Chen

**Affiliations:** 1 State Key Laboratory of Analytical Chemistry for Life Sciences and Collaborative Innovation Center of Chemistry for Life Sciences, State Key Laboratory of Pharmaceutical Biotechnology, School of Life Sciences, Nanjing University, Nanjing 210023, P. R. China; 2 Department of Orthopaedics, Jinling Hospital, School of Medicine, Nanjing University, Nanjing 210002, P. R. China

## Abstract

Osteosarcoma (OS) is the most common primary bone malignancy and remains a leading cause of cancer-related deaths in adolescents. Emerging evidence indicates that microRNAs (miRNAs) are correlated with clinical and biological characteristics of OS. However, the involvement of miR-199a-5p in OS development remains unclear. In this study, we examined the function of miR-199a-5p *in vitro* and *in vivo*. The results showed that miR-199a-5p was significantly up-regulated in OS patient tissues and cells. The inhibition of miR-199a-5p led to a significant decrease in cell proliferation and tumour growth. We further demonstrated that miR-199a-5p could directly bind to the 3′UTRs of the mRNA of both PIAS3 and p27 and mediate a decrease in the protein levels of PIAS3 and p27, thereby stimulating STAT3 activation and cell cycle progression in OS cells. Rescue experiments of PIAS3 and p27 further revealed that PIAS3 and p27 were functional targets of miR-199a-5p. Moreover, enhancing the expressions of both PIAS3 and p27 using miR-199a-5p-targeted inhibitors in an OS xenograft model was shown to be a promising approach for OS clinical therapy. Our findings indicate that the pathway of miR-199a-5p targeting both PIAS3 and p27 is a possible mechanism that contributes to tumour growth in OS.

As the most common primary bone malignancy, osteosarcoma (OS) is the leading cause of cancer-related deaths among children and young adolescents, especially those aged of 15 to 19. Although the precise pathogenic mechanism of OS remains unclear, it is widely accepted that factors such as genetic susceptibility, environmental ionizing radiation and body lesions, among others, may lead to OS. Among these pathogenic factors, genetic mutations and transcription regulation disorders can trigger consistent cell proliferation and thereby accelerate OS development^1^. Although significant improvements in OS treatments such as chemotherapy and radiotherapy have been made in the past several decades, the prognosis for osteosarcoma patients still remains poor^2^. Therefore, elucidating the molecular mechanism underlying OS will contribute to the development of effective strategies for OS treatment and prognosis.

MicroRNAs (miRNAs) are small noncoding RNAs that have been studied extensively in human tumours and acting as oncogenes and tumor suppressors. The fact that microRNA (miRNA) expression fingerprints are correlated with clinical and biological characteristics in tumours raises the potential that a small miRNA molecule could tell a big story in tumour development^3^,^4^. Emerging evidence shows that a variety of miRNAs are aberrantly expressed in the serum and tissues of OS patients, suggesting that miRNAs may play critical roles in controlling the progression of OS. Jones *et al*. identified a miRNA signature based on the comparison of 18 pretreatment biopsy samples from OS tissues with 16 control samples from healthy bone tissues. The signature included increased expression of miR-181a, miR-181b, and miR-181c and reduced expression of miR-16, miR-29b, and miR-142-5p^5^. Duan *et al*. found that miR-199a-3p, miR-127-3p, and miR-376c were significantly decreased whereas miR-151-3p and miR-191 were increased in osteosarcoma cell lines in comparison with osteoblasts^6^. However, the biological functions of most of the differentially expressed miRNAs in OS are still unclear. Osaki *et al*. explored the correlation of the down-regulation of miR-143 with the lung metastasis of osteosarcoma cells by comparing the parental osteosarcoma cell lines HOS with its subclone osteosarcoma cell lines 143B^7^. miR-199a-3p was reported to play an important role in OS cell proliferation and migration through the down-regulation of mTOR, STAT3 and CD44^6^,^8^. Additionally, Zhu *et al*. reported that miR-29b acted as a tumour suppressor by targeting CDK6 in OS cells^9^. Recently, we found that the serum miR-199a-5p concentration was significantly higher in 60 osteosarcoma patients before surgery than in 60 healthy individuals, and that the serum miR-199a-5p level was significantly decreased in 28 patients after surgery compared with the 60 preoperative patients^10^. However, the biological functions of miR-199a-5p in OS remain unclear. The investigation into the effects of miR-199a-5p and its targets in OS may lead to new perspectives for clinical trials of gene therapy.

Evidence has revealed that the activated STAT3 molecule can mediate cellular transformation by becoming phosphorylated (p-STAT3), and the persistently activated STAT3 in some circumstances can participate in cell proliferation and promote tumour progression^11^,^12^. Moreover, several reports have shown that the overexpression of p-STAT3 is associated with poor prognosis and that STAT3 may be a prognostic indicator for osteosarcoma^13^,^14^,^15^. As a specific protein inhibitor of activated STAT3, PIAS3 can interfere with the phosphorylation of STAT3 and inhibit STAT3-mediated gene activation, thereby influencing cell proliferation by regulating proliferation-associated genes, such as PCNA and KI67^16^,^17^. However, the role of PIAS3 in OS is unclear, and whether there are aberrantly expressed miRNAs involved in the regulation of PIAS3 in OS has not been thoroughly studied. P27 (also known as CDKN1B or Kip1) is a cyclin-dependent kinase inhibitor that plays an important role in regulating cell proliferation and the cell cycle, making it a strong candidate as a target to consider in cancer therapy^18^,^19^. Thomas *et al*. found that the loss of p27 expression was correlated with dedifferentiation in high-grade human osteosarcomas and worse prognosis^20^. Nevertheless, the mechanism of the loss of p27 expression in OS and whether the loss of p27 results from miRNA regulation are still unknown.

In this study, we investigated the role of miR-199a-5p in the OS-derived Saos-2 and MNNG/HOS cell lines and in an immunodeficient mouse xenograft model of OS. The influence of miR-199a-5p on cell proliferation and cell cycle progression through its potential targets of PIAS3 and p27 was systematically investigated. Additionally, the role of miR-199a-5p was further evaluated in an OS xenograft model by the intratumoural administration of an anti-miR-199a-5p oligonucleotide (miR-199a-5p AMO).

## Results

### miR-199a-5p is up-regulated in human OS tissues and cell lines

**Figure 1:**
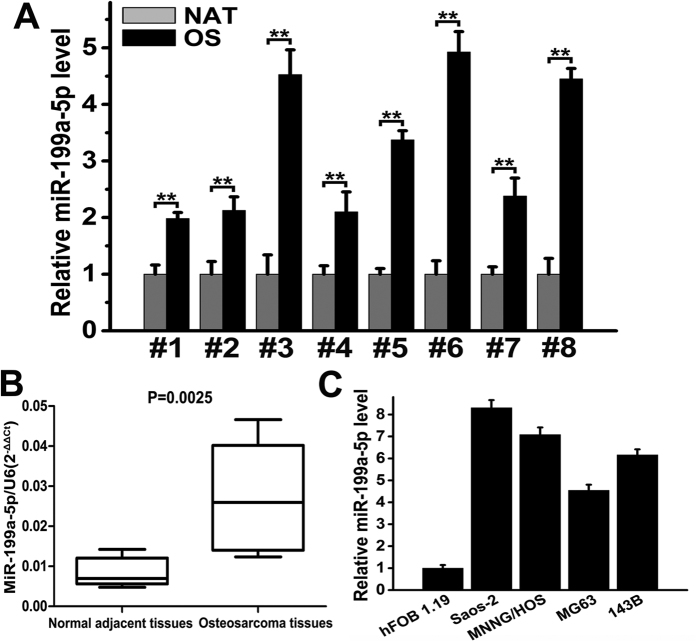
miR-199a-5p is up-regulated in human osteosarcoma tissues and cell lines. (**A**) The relative miR-199a-5p expression in each human osteosarcoma (OS) tissue compared with its paired normal adjacent tissue (NAT) shown as histograms. (**B**) The overall miR-199a-5p expression in the eight human OS and NAT samples shown as box-and-whiskers plots (P = 0.0025). (**C**) The relative miR-199a-5p expression in four human osteosarcoma cell lines (Saos-2, MNNG/HOS, MG63 and 143B) compared with the human osteoblast cell line hFOB 1.19. miR-199a-5p expression was analysed using qRT-PCR, and U6 snRNA was used as an internal control. The data are expressed as the means ± SEM, **P < 0.01.

Previously, we demonstrated that serum miR-199a-5p is up-regulated in OS patients using a high-throughput TaqMan low-density qPCR array (TLDA) and qRT-PCR assays. Here, we performed qRT-PCR assays on eight pairs of OS and normal adjacent tissue (NAT) samples to quantify the expression of miR-199a-5p. As shown in Fig. 1A and B, the qRT-PCR analysis revealed that the miR-199a-5p level was significantly increased in OS samples. The overall level of miR-199a-5p increased 3.2-fold in all osteosarcoma samples compared with the NAT samples, indicating that the up-regulation of miR-199a-5p is a frequent event in osteosarcoma. We next examined miR-199a-5p expression in four OS cell lines (Saos-2, MNNG/HOS, MG63 and 143B) as well as in hFOB 1.19 cells by qRT-PCR. miR-199a-5p was expressed in all five cell lines, and the levels of miR-199a-5p in the four OS cell lines were higher than that in the hFOB 1.19 cells. Among the four OS cell lines, Saos-2 and MNNG/HOS expressed a relatively high level of miR-199a-5p (Fig. 1C). Therefore, we used Saos-2 and MNNG/HOS cells to investigate the effect of miR-199a-5p on cell proliferation and tumour growth.

### The effects of miR-199a-5p on cell proliferation and tumour growth in OS

**Figure 2:**
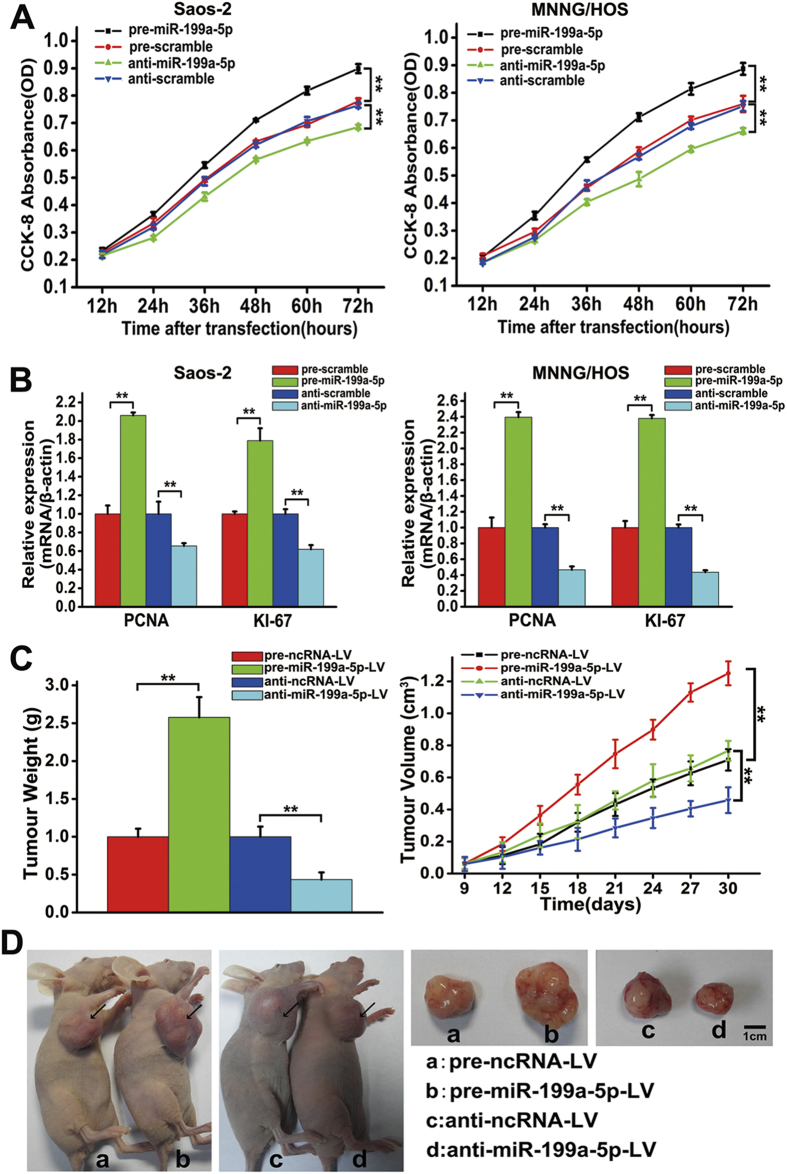
miR-199a-5p promotes OS cell proliferation *in vitro* and tumour growth *in vivo*. (**A**) Growth curves of Saos-2 (left) and MNNG/HOS cells (right) after transfection with pre/anti-miR-199a-5p or the corresponding control. The measurements of the cell growth rate were obtained using a CCK-8 kit. The experiments were repeated three times. (**B**) Relative mRNA levels of KI-67 and PCNA in Saos-2 (left) and MNNG/HOS cells (right) after transfection with pre/anti-miR-199a-5p or the corresponding control. (**C**) The weight (left) and volume (right) of the xenograft tumours in nude mice derived from the subcutaneous implantation of MNNG/HOS cells stably expressing or inhibiting miR-199a-5p or their control cells. (**D**) Representative photographs of the tumour-bearing mice and the excised tumours at 30 days post-implantation. The data are expressed as the means ± SEM. **P < 0.01.

Considering that miR-199a-5p was up-regulated in OS tissues, we next examined the effect of miR-199a-5p on cell proliferation *in vitro*. As shown in Fig. 2A, the over-expression of miR-199a-5p significantly promoted cell proliferation, while the knock-down of miR-199a-5p significantly inhibited the proliferation of both Saos-2 and MNNG/HOS cells. QRT-PCR results showed that the mRNA levels of PCNA and KI-67 were increased when miR-199a-5p was over-expressed, while the knock-down of miR-199a-5p reduced the mRNA levels of PCNA and KI-67 (Fig. 2B). These results indicated that the dysregulated expression of miR-199a-5p might have an influence on OS tumour growth *in vivo*. Saos-2 cells were not tumourigenic in immunosuppressed mice, while MNNG/HOS cells were tumourigenic within 21 days at 100% frequency in nude mice that were inoculated subcutaneously with 10^7^ cells. To examine the effect of miR-199a-5p on tumour growth *in vivo*, we used MNNG/HOS cells to generate stable cell lines expressing or inhibiting miR-199a-5p. As shown in Supplementary Fig. S1A, the expression of GFP or mCherry could be observed in the sorted MNNG/HOS cells by a Nikon confocal microscope, indicating the successful generation of stable cell lines. Furthermore, the level of miR-199a-5p in sorted MNNG/HOS cells was detected by qRT-PCR. The result indicated that the expression of miR-199a-5p increased 15.2-fold in pre-miR-199a-5p-LV cells compared with the pre-ncRNA-LV cells, while the level of miR-199a-5p decreased 73.6% in the anti-miR-1992-5p-LV cells compared with the anti-ncRNA-LV cells (Supplementary Fig. S1B), suggesting that the sorted MNNG/HOS cells could be used as stable cell lines expressing or inhibiting miR-199a-5p for the next study in the immunodeficient mouse xenograft tumour model. After 4 groups of nude mice (7 mice/group) were subcutaneously implanted with the sorted cells, we observed that the size of tumours in the pre-miR-199a-5p-LV group was significantly larger than those in the pre-ncRNA-LV group at each time point, whereas the size of tumours in the anti-miR-199a-5p-LV group exhibited the opposite trend (Fig. 2C). The excised tumours from the pre-miR-199a-5p-LV group were 1.57-fold heavier than those from the pre-ncRNA-LV group at 30 days post-implantation, whereas the tumours from the anti-miR-199a-5p-LV group weighted 56.5% less than those from the anti-ncRNA-LV group (Fig. 2C). These results suggested that the over-expression of miR-199a-5p enhanced the growth of MNNG/HOS cell-induced tumours, while the knock-down of miR-199a-5p inhibited the tumour growth *in vivo*. Representative photographs of tumour-bearing mice and the corresponding tumours from each group are shown in Fig. 2D.

### miR-199a-5p up-regulation suppresses PIAS3 expression and causes STAT3 activation

In OS, the up-regulation of miR-199a-5p could promote cell proliferation and tumour growth. However, the regulatory mechanism for this is not clear yet. Therefore, we performed bioinformatics analyses to search for miR-199a-5p-targeted genes. TargetScan, Miranda, and PICTAR5 were independently used to predict the target genes. Through the combination of these bioinformatics prediction methods and a literature review, we found that PIAS3 (a specific inhibitor of activated STAT3) and p27 (a negative cell-cycle regulator) are likely targets of miR-199a-5p. In the following experiments, we focused attention on the interaction between PIAS3/p27 and miR-199a-5p in the development of OS.

**Figure 3:**
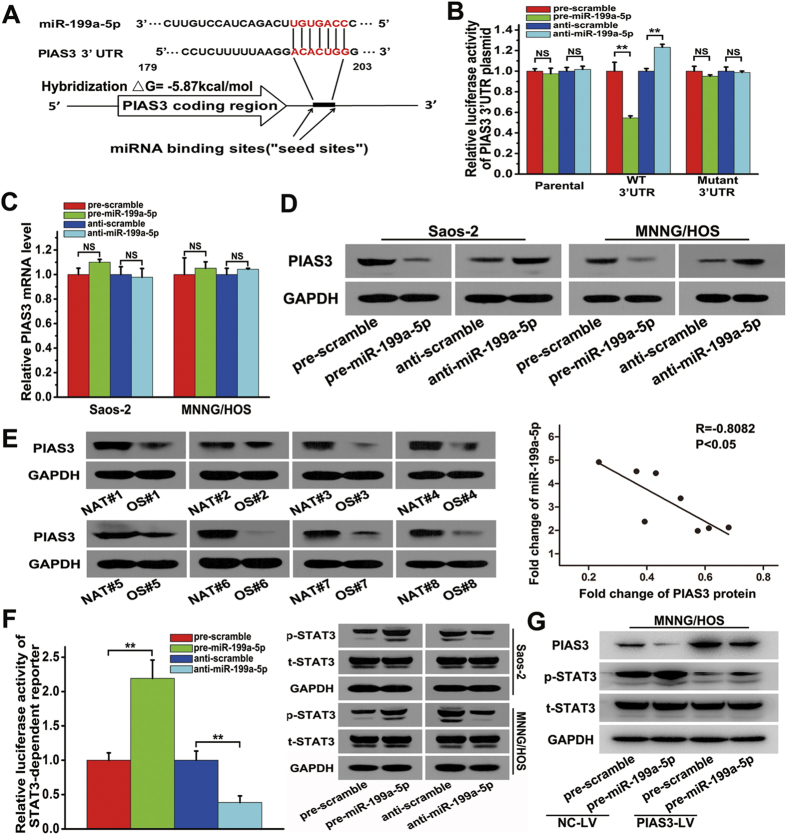
Identification of PIAS3 as a target of miR-199a-5p and the activation of STAT3 by miR-199a-5p. (**A**) TargetScan prediction software identified one seed match for miR-199a-5p in the 3′UTR of PIAS3; the predicted seed-recognition site in the PIAS3 mRNA sequence and the corresponding miR-199a-5p sequence are marked in red. (**B**) The relative luciferase activity of the PIAS3 3′-UTR reporter plasmid was assayed in MNNG/HOS cells after transfection with pre/anti-miR-199a-5p or the corresponding control. (**C**) The relative mRNA level of PIAS3 in Saos-2 (left) or MNNG/HOS cells (right) after transfection with pre/anti-miR-199a-5p or the corresponding control. (**D**) The protein level of PIAS3 in Saos-2 (left) or MNNG/HOS cells (right) after transfection with pre/anti-miR-199a-5p or the corresponding control. (**E**) The protein level of PIAS3 in the eight pairs of NAT and OS samples, as determined using western blotting (left). Pearson’s correlation scatter plot comparing the fold changes in the expression of miR-199a-5p and the PIAS3 protein in OS patients (right). (**F**) The relative luciferase activity of the STAT3-dependent reporter construct in MNNG/HOS cells (left) and the protein levels of p-STAT3 and total STAT3 in Saos-2 and MNNG/HOS cells (right) after transfection with pre/anti-miR-199a-5p or the corresponding control. (**G**) The protein levels of PIAS3, p-STAT3 and total STAT3 in MNNG/HOS cells stably over-expressing PIAS3 and the control cells after transfection with pre-miR-199a-5p or the corresponding control. One representative image of three reproducible results is shown for the western blot assay. The data are expressed as the means ± SEM, **P < 0.01.

Accordingly, the over-expression of miR-199a-5p suppressed approximately 45.5% of the luciferase activity of the PIAS3 3′UTR reporter construct, while the inhibition of miR-199a-5p resulted in a 23.2% increase in reporter activity compared with the control. Mutagenesis of the predicted miR-199a-5p binding sites restored the luciferase expression, thereby confirming the specificity of the interaction between miR-199a-5p and the PIAS3 3′UTR (Fig. 3A and B). However, the mRNA level of PIAS3 did not appear to change in response to the over-expression and knock-down of miR-199a-5p (Fig. 3C). Next, we used western blotting to examine the PIAS3 protein level after the over-expression and knock-down of miR-199a-5p in Saos-2 or MNNG/HOS cells (Fig. 3D). Compared with the control, the over-expression of miR-199a-5p resulted in a significant decrease in the expression of PIAS3, while the knock-down of miR-199a-5p led to an increase in PIAS3 expression. The above results demonstrated that miR-199a-5p regulated PIAS3 expression only via a translational inhibition mechanism, rather than by affecting its mRNA stability. In addition, as shown in Fig. 3E, the PIAS3 protein level was down-regulated in eight OS samples compared with NAT samples, and the overall PIAS3 level in OS samples was 58.1% lower than that of NAT samples. The value of R = −0.8082 in Fig. 3E suggested an inverse correlation between the expression of miR-199a-5p and the PIAS3 protein.

To study the effect of miR-199a-5p targeting PIAS3, we analysed the expression of p-STAT3 and total STAT3 protein using western blotting after transfection with pre/anti-miR-199a-5p. As shown in Fig. 3F, miR-199a-5p had no obvious effect on the total STAT3 expression; however, it did enhance the phosphorylation of STAT3. The over-expression of miR-199a-5p led to an increase in p-STAT3, and the knock-down of miR-199a-5p decreased p-STAT3 expression in Saos-2 or MNNG/HOS cells. In addition, a luciferase reporter construct with STAT3-dependent transcriptional activity was integrated into MNNG/HOS cells, which were used to further examine the effects of miR-199a-5p targeting PIAS3 (as shown in Fig. 3F). The over-expression of miR-199a-5p led to a 1.2-fold increase in STAT3-dependent luciferase activity, and the knock-down of miR-199a-5p resulted in an approximately 61.4% reduction of the STAT3-dependent luciferase activity in MNNG/HOS cells compared with the control cells. Thereafter, we performed rescue experiments to investigate the effect of miR-199a-5p in MNNG/HOS cells stably over-expressing PIAS3. As shown in Fig. 3G, the protein level of p-STAT3 was obviously reduced in PIAS3-over-expressing cells (PIAS3-LV) after transfection with pre-miR-199a-5p, compared with their control cells (NC-LV). Together, these data indicate that the over-expression of miR-199a-5p could promote the phosphorylation of STAT3 by down-regulating the expression of PIAS3.

### miR-199a-5p targets p27 and influences the cell cycle

**Figure 4:**
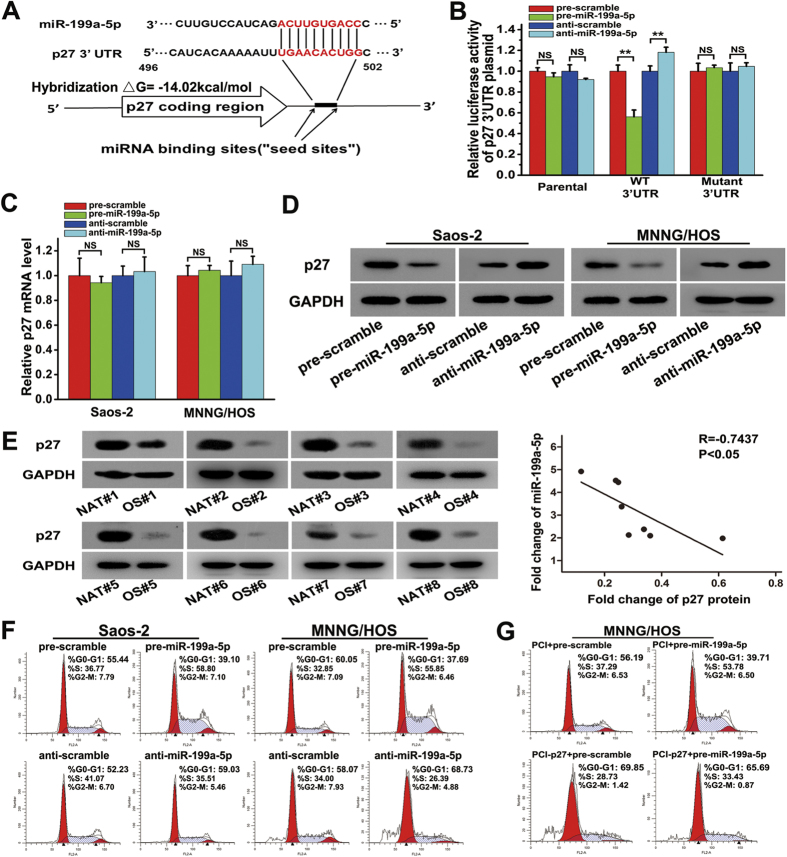
miR-199a-5p targets p27 and induces the G1-to-S cell-cycle transition. (**A**) TargetScan prediction software identified one seed match for miR-199a-5p in the 3′UTR of p27; the predicted seed-recognition site in the p27 mRNA sequence and the corresponding miR-199a-5p sequence are marked in red. (**B**) The relative luciferase activity of the p27 3′-UTR reporter plasmid was assayed in MNNG/HOS cells after transfection with pre/anti-miR-199a-5p or the corresponding control. (**C**) The relative level p27 mRNA in Saos-2 (left) and MNNG/HOS cells (right) after transfection with pre/anti-miR-199a-5p or the corresponding control. (**D**) The protein level of p27 in Saos-2 (left) or MNNG/HOS cells (right) after transfection with pre/anti-miR-199a-5p or the corresponding control. (**E**) The protein expression of p27 in the eight pairs of NAT and OS samples (left). Pearson’s correlation scatter plot comparing the fold changes in the expression of miR-199a-5p and the p27 protein in OS patients (right). (**F**) Cell cycle analysis of Saos-2 and MNNG/HOS cells after transfection with pre/anti-miR-199a-5p or the corresponding control. (**G**) Cell cycle analysis of MNNG/HOS cells after transfection with pre-scramble plus the PCI plasmid, pre-miR-199a-5p plus the PCI plasmid, pre-scramble plus the PCI-p27 plasmid or pre-miR-199a-5p plus the PCI-p27 plasmid for 48 hours. The data are expressed as the means ± SEM, **P < 0.01. NS: no significant change.

It was confirmed that PIAS3 could be targeted by miR-199a-5p, while it was not clear whether the predicted p27 could be another target of miR-199a-5p. As shown in Fig. 4A and B, the over-expression of miR-199a-5p suppressed approximately 43.8% of the luciferase activity of the p27 3′UTR construct, while the inhibition of miR-199a-5p resulted in an 18.2% increase in reporter activity compared with the control. Mutagenesis of the predicted miR-199a-5p binding sites restored the luciferase expression. Nevertheless, the results in Fig. 4C show that the mRNA levels of p27 were not affected by the over-expression and knock-down of miR-199a-5p. Next, western blotting was used to examine the p27 protein level after the over-expression and knock-down of miR-199a-5p in Saos-2 or MNNG/HOS cells. Compared with the control, the over-expression of miR-199a-5p resulted in a significant decrease in the expression of p27, while the knock-down of miR-199a-5p led to an increase in p27 expression (Fig. 4D). The correlation between miR-199a-5p and p27 was further examined in OS tissues. As shown in Fig. 4E, the p27 protein was down-regulated in OS samples compared with NAT samples. The overall p27 protein level in OS samples was 74.3% lower than that in NAT samples. The value of R = −0.7437 suggested an inverse correlation between the expression of miR-199a-5p and the p27 protein level in OS samples. Accordingly, these results revealed that miR-199a-5p could regulate p27 expression only via a translational inhibition mechanism.

As a tumour suppressor, p27 influences cell cycle progression by negatively regulating the activity of cyclin-dependent kinases. Flow cytometry was used to examine whether miR-199a-5p influences the cell cycle in Saos-2 and MNNG/HOS cells. As shown in Figs 4F and S3, transfection with pre-miR-199a-5p in Saos-2 cells for 48 hours resulted in a distinct decrease in the G1-phase cell population (55.44% vs. 39.10%) and an increase in the S-phase cell population (36.77% vs. 58.80%), compared with the control cells. In contrast, in anti-miR-199a-5p-transfected cells, an increase in the G1-phase cell population (52.33% vs. 59.03%) and a decrease in the S-phase cell population (41.07% vs. 35.51%) were detected compared with the corresponding control cells. Similar results were observed in MNNG/HOS cells. Moreover, rescue experiments were performed to study the effect of miR-199a-5p on the cell cycle in MNNG/HOS cells over-expressing p27. As shown in Figs 4G and S5, an obvious decrease in the S-phase cell population (53.78% vs. 33.43%) was detected after co-transfection of pre-miR-199a-5p and the PCI-p27 plasmid for 48 hours, while the protein level of p27 increased compared with cells transfected with pre-miR-199a-5p plus PCI (Fig. S4), suggesting that the ectopic over-expression of p27 was able to delay the G1-S phase transition of the cell cycle caused by miR-199a-5p over-expression. Taken together, these data indicate that miR-199a-5p functions by targeting p27 in OS.

### Therapeutic effect of miR-199a-5p inhibitors *in vivo*

**Figure 5:**
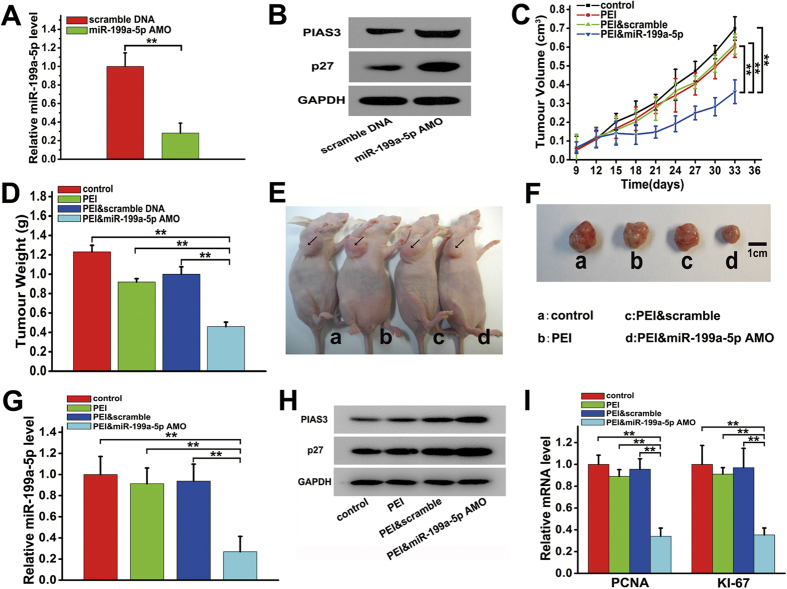
Anti-tumour effects of an anti-miR-199a-5p oligonucleotide (miR-199a-5p AMO) *in vivo*. (**A**) The relative expression of miR-199a-5p in MNNG/HOS cells 24 hours after transfection with the miR-199a-5p AMO or scramble DNA. (**B**) The protein level of PIAS3 and p27 in MNNG/HOS cells 24 hours after transfected with miR-199a-5p AMO or scramble DNA. (**C**) The volumes of the xenograft tumours in nude mice determined at 9, 12, 15, 18, 21, 24, 27, 30, and 33 days tumours transplantation, followed by intratumoral treatment with miR-199a-5p AMO on day 9 after tumours transplantation. (**D**) The weights of the xenograft tumours in nude mice measured at 33 days after tumours transplantation, followed by intratumoral treatment with miR-199a-5p AMO on day 9 after tumours transplantation. (**E**) A representative photograph of tumour-bearing mice 33 days after tumours transplantation and intratumoral injection with miR-199a-5p AMO. (**F**) A representative photograph of tumours excised from tumour-bearing mice, 33 days after tumours transplantation and intratumoral injection with miR-199a-5p AMO. (**G**) QRT-PCR analysis of miR-199a-5p and (**I**) QRT-PCR analysis of PCNA and KI-67 in the xenograft tumours in nude mice 33 days after tumours transplantation and intratumoral injection with miR-199a-5p AMO. The data are expressed as the means ± SEM, **P < 0.01. (**H**) Western blot analysis of PIAS3 and p27 in the xenograft tumours in nude mice 33 days after tumours transplantation and intratumoral injection with miR-199a-5p AMO.

Given that the dysregulated expression of miR-199a-5p could result in significant changes in the expression of PIAS3 and p27 (Figs 3D and 4D and Fig. S2), we attempted to use miR-199a-5p as a therapeutic target in OS. We designed a miR-199a-5p AMO to inhibit miR-199a-5p expression in the nude mouse xenograft model of human osteosarcoma. As shown in Fig. 5A and B, when MNNG/HOS cells were transfected with the miR-199a-5p AMO, the miR-199a-5p level declined by 67.3%, and the levels of PIAS3 and p27 were increased by approximately 53.2% and 78.3%, respectively, compared to transfection with the scrambled DNA. The anti-tumour activity of the miR-199a-5p AMO was then assessed *in vivo*. After intratumoural treatment with PEI and miR-199a-5p AMO complexes when the tumour volumes reached a size of approximately 100 mm^3^ in the OS xenograft model, the mice showed greatly attenuated tumour growth, reduced tumour volume, and decreased tumour weight, while no significant differences in tumour growth were observed between the mice treated with PEI and scrambled DNA complexes or PEI alone and the control group (Fig. 5C and D). As shown in Fig. 5D, the tumour weight in the PEI & miR-199a-5p AMO group was 54.0% less than that in the PEI & scramble DNA group. Representative photographs of tumour-bearing mice and the corresponding tumours from each group are shown in Fig. 5E and F. Moreover, after treatment with the miR-199a-5p AMO, the levels of miR-199a-5p (Fig. 5G), KI-67 and PCNA (Fig. 5I) significantly declined, while the protein levels of p27 and PIAS3 (Fig. 5H) significantly increased, suggesting that an inhibitor targeting miR-199a-5p could remarkably suppress tumour growth in OS.

## Discussion

In the present study, we demonstrated that miR-199a-5p was up-regulated in OS patient tissues and cell lines and might promote OS cell proliferation. This result was further supported by the findings showing that the over-expression of miR-199a-5p induced tumour growth and the knock-down of its expression had the opposite effect in a nude mouse xenograft model of OS. Previous studies have demonstrated that miR-199a-5p is involved in the progression of several cancers, including hepatocellular carcinoma (HCC), small cell carcinoma of the cervix (SCCC), gastric cancer, multiple myeloma and melanoma^21^,^22^,^23^,^24^,^25^. Interestingly, miR-199a-5p exerts diverse and conflicting biological effects in several cancers depending on the specific cell type and context. For example, miR-199a-5p is up-regulated in gastric cancer and melanoma, while it may act as a tumour suppressor in HCC, SCCC and multiple myeloma. Our results not only suggested an oncogenic role of miR-199a-5p in the development of OS but also provided a potential gene therapeutic target for OS.

In addition, PIAS3 and p27 were identified as the dual targets of an oncogenic miR-199a-5p in OS. Based on three bioinformatics analyses, nineteen genes were predicted to be targets of miR-199a-5p, as shown in Supplementary Table 3. The first eight of the nineteen targets, PIAS3, p27, RAB10, DBF4, DUSP14, RBPMS, PDPN and CLTC, were considered to be closely associated with cancer progression after a literature review. Among them, RAB10, a member of the RAS superfamily, might function as an oncogenic small GTPase in OS^26^. Kunita A, *et al*. found that PDPN was highly expressed in metastatic osteosarcoma and could promote cell migration in OS^27^. The oncogenic role of CLTC in OS was suggested because it was related to IGF1R endocytosis-triggering downstream signalling activation and cell proliferation^28^. Moreover, the three genes DBF4, DUSP14 and RBPMS have not been characterized in OS but have been reported to tend to play carcinogenic roles during the tumour development^29^,^30^,^31^. Based on the fact that miR-199a-5p was up-regulated in OS patient tissues and cell lines, we concluded that the six genes RAB10, PDPN, CLTC, DBF4, DUSP14 and RBPMS could not be the targets of miR-199a-5p in OS. Additionally, Klotho and ApoE have been reported as the targets of miR-199a-5p in gastric cancer and melanoma, respectively^23^,^25^. Since their functions in OS have not yet been elucidated, whether Klotho and ApoE could be regulated by miR-199a-5p in OS needs further investigation. On the basis of the inverse correlation between the expression of miR-199a-5p and the protein levels of PIAS3 and p27 in OS patient tissues and other evidence, we considered that the pathway of miR-199a-5p targeting both PIAS3 and p27 is a possible mechanism that contributes to tumour growth in OS.

Our data in this study clearly demonstrated that miR-199a-5p could activate STAT3 signalling by targeting PIAS3 and lead to significantly increased levels of PCNA and KI67 in OS. In addition to miR-199a-5p in OS, other miRNAs that could target PIAS3 in tumours have been reported. miR-18a, miR-21 and miR-125b have been shown to act as oncogenic miRNAs and negatively regulate PIAS3 in gastric carcinogenesis, multiple myeloma and glioblastoma stem cells, respectively^32^,^33^,^34^. Among these three miRNAs, miR-125b was found to be down-regulated in OS^35^,^36^, indicating that miR-125b could not regulate PIAS3 in OS. The over-expression of miR-18a has been reported in OS cell lines^37^. miR-21 was also identified to be highly expressed in OS cells and patient tissues and functions by targeting RECK and PTEN^38^,^39^. In this study, we found that the fold increases in the expression of miR-18a and miR-21 were similar to that of miR-199a-5p in OS patient tissues (data not shown), but the serum levels of miR-18a and miR-21 in OS patients did not show significant differences^10^. Whether miR-18a, miR-21 and miR-199a-5p could convergently target PIAS3 in OS needs further study. Furthermore, we validated that miR-199a-5p could target p27 and affect cell cycle progression in OS. Our findings elucidated that it is likely miR-199a-5p, acting as an oncogenic miRNA, that could lead to the loss of p27 and regulate the cell proliferation and cell cycle in OS. Cao *et al*. and Wang *et al*. revealed that the expression of p27 was negatively regulated by miR-802 and miR-25^40^,^41^, thus illustrating that p27 might be regulated by several miRNAs simultaneously in OS. Together, our data indicate that miR-199a-5p as an oncogenic miRNA can remarkably promote OS progression by targeting both PIAS3 and p27.

For newly diagnosed osteosarcoma patients, in order to clear the subclinical lesions more thoroughly and reduce the risk of recurrence, the current treatment usually consists of the combination of chemotherapy and radiation to inhibit growth before surgery, which inevitably results in severe side effects^2^. Since miRNAs can produce global effects on a series of genes, miRNAs might be ideal agents for a combined tumour therapy alternative to chemotherapy and radiation. Recent studies have shown that the delivery of miR-143 by atelocollagen can significantly suppress the metastasis of osteosarcoma^7^, and various strategies for the delivery of miR-34 mimics exhibit remarkable inhibition effects in mouse models of neuroblastoma, non-small-cell lung cancer (NSCLC) and colon cancer, among others. In addition, phase I clinical trials of a liposome-based miR-34 drug for hepatocellular carcinoma patients are currently under way^42^,^43^. Because cell proliferation is a prerequisite for metastasis, appearing as the most common cause of OS recurrence and death, we speculate that therapies targeting miR-199a-5p might be useful for OS treatment due to the dual targeting of miR-199a-5p to PIAS3 and p27. In this study, our results further revealed that reducing the miR-199a-5p level by biologically stable antisense oligonucleotides of miR-199a-5p significantly inhibited the growth of osteosarcoma tumours in nude mice, indicating that the administration of miR-199a-5p inhibitors could complement or improve current OS therapeutic strategies.

In conclusion, we identified that miR-199a-5p was significantly up-regulated in OS patient tissues and that its expression was inversely correlated with PIAS3 and p27 protein expression in OS. miR-199a-5p could stimulate osteosarcoma cell proliferation and tumour growth *in vitro* and *in vivo*. The possible mechanism was further studied in OS cells, and the targeting of PIAS3 and p27 by miR-199a-5p was identified. Additionally, a miR-199a-5p-targeted inhibitor could significantly enhance PIAS3 and p27 expression and ultimately suppress the tumour growth in a xenograft model of OS, which represents a potential therapeutic approach that may be valuable for OS therapy.

## Materials and Methods

### Clinical samples, cell lines and chemical reagents

Surgically resected paired osteosarcoma (OS) and normal adjacent tissues (NAT) were obtained from patients who underwent radical resection at Jinling Hospital (Nanjing, P. R. China) from 2012 to 2015. The surgically removed tissues were quickly frozen in liquid nitrogen until analysis. All protocols concerning the use of patient samples in this study were approved by the Medical Ethics Committee of the Affiliated Jinling Hospital of Nanjing University (Nanjing, China). A signed informed consent was obtained from each patient. And the clinical information of these patients is listed in Supplementary Table 1. The investigations were conducted according to the Declaration of Helsinki principles.

Cells were maintained in 5% CO_2_ at 37 °C in a humidified atmosphere in McCoy’s 5A medium (Saos-2), EMEM (MNNG/HOS, MG63) or DMEM (143B, hFOB 1.19) supplemented with 10% FBS (Life Technologies, Grand Island, NY, USA). All cell lines were obtained from the Institute of Cell Biology at the Chinese Academy of Sciences (Shanghai, P. R. China). All chemical reagents were purchased from Sigma-Aldrich (St. Louis, MO, USA).

### RNA extraction and quantitative real-time PCR (qRT-PCR) assays

Total RNA from the cultured cells and tissues was prepared using the TRIzol reagent (Life Technologies). The qRT-PCR assays were performed using the SYBR PrimeScript™ miRNA RT-PCR Kit (Takara, Shiga, Japan) to examine miRNA levels or using the One Step SYBR PrimeScript™ RT-PCR Kit (Takara) to analyse gene expression according to the manufacturer’s protocols. The level of U6 snRNA was used as an internal control for miRNA expression, and the expression of genes was normalized to the expression of β-actin. All primer sequences for the qRT-PCR analysis of miRNAs and genes are listed in Supplementary Table 2.

### Cell transfection/infection assays

Saos-2 or MNNG/HOS cells were transfected with precursor oligonucleotides (pre-miR-199a-5p), antisense oligonucleotides (anti-miR-199a-5p) or their corresponding controls (pre-scramble or anti-scramble) (Life Technologies) using the Lipofectamine 2000 transfection reagent (Life Technologies) according to the manufacturer’s instructions. In general, the cells were collected for RNA assays 24 hours after transfection or for protein analysis 48 hours after transfection.

To obtain MNNG/HOS cells stably expressing or inhibiting miR-199a-5p, MNNG/HOS cells were infected with pre/anti-miR-199a-5p-LV (lentivirus carrying either pre-miR-199a-5p precursor or anti-miR-199a-5p inhibitor and an eGFP or mCherry fluorescent tag, respectively) or infected with pre/anti-NC-LV (the corresponding control lentivirus carrying a pre-noncoding/anti-noncoding sequence and an eGFP/mCherry fluorescent tag) (GeneCopoeia, Guangzhou, China) in the presence of 8 μg/ml polybrene (GeneCopoeia) for 12 hours. All lentiviral constructs also contained a puromycin resistance sequence for drug screening. Three days after infection, the cells were then cultured in medium with 10 μg/ml puromycin (Sigma-Aldrich). Additionally, the MNNG/HOS cells stably expressing PIAS3 (PIAS3-LV) and their control cells (NC-LV) were sorted based on puromycin resistance after being infected with PIAS3-LV (lentivirus carrying the coding sequence of PIAS3 and containing a puromycin resistance sequence for drug screening) or NC-LV (the corresponding control lentivirus carrying a noncoding sequence and a puromycin resistance sequence) (GeneCopoeia).

### Cell proliferation assay

The cell proliferation assay was performed as previously described^44^. Briefly, Saos-2 or MNNG/HOS cells with over-expression or knocked down-expression of miR-199a-5p were seeded onto 96-well plates at a density of 6 × 10^3^ cells per well. The number of viable cells at 12, 24, 36, 48, 60 and 72 hours was determined using WST-8 staining with a Cell Counting Kit-8 (CCK-8, Dojindo, Tokyo, Japan) according to the manufacturer’s instructions. In addition, the mRNA levels of the proliferation markers PCNA and KI-67 were used to assess the growth of Saos-2 or MNNG/HOS cells after transfection with pre-/anti-miR-199a-5p.

### The immunodeficient mouse xenograft model of human osteosarcoma

Animal protocols were reviewed and approved by the Animal Care and Use Committee of Nanjing University, and conformed to the Guidelines for the Care and Use of Laboratory Animals published by the National Institutes of Health. Four-week-old, thymic BALB/c male, nude (nu/nu) mice were obtained from the Laboratory Animal Center of Nanjing University and maintained under pathogen-limited conditions. The animals were divided equally into 4 groups (7 mice per group) and 1 × 10^7^ viable MNNG/HOS cells stably expressing/inhibiting miR-199a-5p or their control cells were injected subcutaneously into the right flanks of the mice. After the subcutaneous implantation of cells, the animals were observed daily for tumour growth and subcutaneous tumours were measured on days 9, 12, 15, 18, 21, 24, 27 and 30. The tumour volume was calculated according to the following equation: V (mm^3^) = D^2^ (mm^2^) × L (mm)/2, where D and L are the smallest and the largest perpendicular tumour diameters, respectively. The mice were sacrificed and photographed at 30 days post-implantation; the xenograft tumours were then excised, photographed and weighed.

### Plasmid constructs and luciferase reporter assay

To construct the luciferase reporter plasmids containing the PIAS3 3′-UTR and p27 3′-UTR, the 3′ untranslated region (3′-UTR) sequences of human PIAS3 and p27, obtained from the GenBank database, were amplified by PCR using a human genomic DNA template made from Saos-2 cells. The reverse primer for the 3′-UTR of PIAS3 was 5′-AGAAGATTGGGAAGGAGGG-3′, and the corresponding forward primer was 5′-AATGTTCTTTAGATGGTGGCA-3′. The sequences of 5′- AGGGCAGTGAGGATAGGTTT-3′ and 5′-ATCGCTGACTTCATGGAATG-3′ were used to amplify the 3′-UTR of p27. The PCR products of the 3′-UTRs of PIAS3 and p27 were inserted into the Spe I/Hind III or Sac I/Hind III sites, respectively, of the p-MIR-reporter plasmid (Promega, Madison, WI, USA), and their efficient insertion was confirmed by sequencing. To construct the PCI plasmid (Life Technologies) carrying the coding sequence of the human p27 gene (PCI-p27), a ClonExpress II One Step Cloning Kit (Vazyme Biotech, Nanjing, China) was used. The reverse primer for the coding sequence of p27 was 5′- TCACTATAGGCTAGCCTCGAGATGTCAAACGTGCGAGTGTCTAA-3′, and the corresponding forward primer was 5′-GCCGCCCGGGTCGACTCTAGATTACGTTTGACGTCTTCTGAGGC-3′.

For the luciferase reporter assays, MNNG/HOS cells were cultured in 6-well plates. In each well, cells at 70–80% confluence were transfected with 1 μg of the firefly luciferase reporter plasmid, 0.5 μg of a β-galactosidase expression vector (Promega) and 100 pmol of pre/anti-miR-199a-5p or pre/anti-scramble using Lipofectamine 2000. The binding-site mutant luciferase reporter plasmids (the binding site ACACTGGG replaced by CCCAGTGT for PAIS3 or the binding site TGAACACTGG replaced by CCAGTGTTCA for p27) were also transfected as a control. The co-transfected β-galactosidase expression vector was used for normalization.

In addition, the luciferase reporter construct pGMLV-STAT3-Lu (carrying seven copies of Stat3-binding sites and a puromycin resistance sequence) was purchased (QianChen Bio-tech, Shanghai, China) in order to measure STAT3-dependent transcriptional activity. MNNG/HOS cells were infected with lentiviruses carrying pGMLV-STAT3-Lu for 12 hours. Three days later, the MNNG/HOS cells that had stably integrated pGMLV-STAT3-Lu were screened in medium containing 10 μg/ml puromycin (Sigma-Aldrich). Thus, for the MNNG/HOS cells with stably integrated pGMLV-STAT3-Lu, the cellular luciferase activity could suggest the STAT3-dependent transcriptional activity. The luciferase reporter assays in the sorted MNNG/HOS cells were performed according to the method in the above paragraph.

### Protein extraction and western blotting

Western blotting was performed as previously described^44^. Protein extracts of cells and tissues were prepared using RIPA lysis buffer (Sigma-Aldrich), and the total protein content was quantified by the BCA protein assay kit (Thermo Scientific, Rockford, IL, USA). The antibodies included PIAS3, p27, p-STAT3 and total-STAT3 (1:1000 dilution, Cell Signaling Technology, Boston, MA, USA). The primary antibodies were incubated overnight at 4 °C followed by a horseradish peroxidase-conjugated goat anti-rabbit IgG (Jackson ImmunoResearch Laboratories, Inc., West Grove, PA, USA). The protein expression was normalized by blotting the same membranes with an antibody against GAPDH (1:10000 dilution, KangCheng Bio-tech, Shanghai, China).

### Cell cycle assay

Saos-2 or MNNG/HOS cells were transfected with 100 pmol of pre-scramble, pre-miR-199a-5p, anti-scramble or anti-miR-199a-5p. At 48 hours post transfection, the cells were collected and washed with PBS. Next, the cells were fixed with 70% ethanol overnight at −20 °C, washed with PBS, resuspended in 400 μl of PBS and then incubated with 100 μg/ml RNase A (Takara) for 30 minutes at 37 °C and with 50 μg/ml propidium iodide (PI) (Sigma-Aldrich) for another 10 minutes. After incubation, the cells were subjected to DNA content analysis using a FACSCalibur system (BD Biosciences, San Jose, CA) and the results were analysed with the ModFit_LT software.

### Therapeutic effects of a miR-199a-5p inhibitor on an immunodeficient mouse OS xenograft model

MiR-199a-5p AMOs with full phosphorothioate linkage were designed and synthesized by SBS Genetech Co., Ltd. (Shanghai, China). A miR-199a-5p AMO with the sequence of 5′-ACAGGTAGTCTGAACACTGG-3′ was selected due to its good inhibition of miR-199a-5p *in vitro*. Polyetherimide (PEI, 25 kDa)/miR-199a-5p AMO complexes were prepared as prevously reported^45^. A solution of the miR-199a-5p AMO (2 mg/ml) was mixed with an equal volume of a PEI aqueous solution (4 mg/ml) for 30 minutes at room temperature for the following therapeutic experiments.

These animal experiments were also approved by the Animal Care and Use Committee of Nanjing University and performed in accordance with the appropriate guidelines as described above. Xenografts of MNNG/HOS cells were initiated by subcutaneous injections of 1 × 10^7^ cells into the right flanks of four nude mice. After 3 weeks, the tumours were dissected and mechanically minced, and a tumour tissue fragment with volume of 3 mm^3^ was transplanted subcutaneously into each nude mouse with a trocar needle. Nine days after the transplantation, when the tumours had grown to approximately 50 mm^3^, the mice were randomized into four groups, a PEI & miR-199a-5p AMO treatment group, PEI & scramble DNA treatment group, or PEI treatment group and a non-treatment (control) group (7 mice per group). Treatments of 100 ug of AMO in a total volume of 50 μl were injected directly into the tumour beginning on day 9 after tumour transplantation and were given once every three days for total eight injections. Tumour volumes were determined at the time of every injection, and the tumours were excised on day 33 after tumour transplantation. Upon termination of the experiment, the mice were sacrificed, and the tumours were removed, photographed, and weighed. The tumour tissue specimens were snap frozen and stored at −70 °C for further analyses.

### Statistical analysis

All experiments were conducted in triplicate and repeated at least three independent times. The data are expressed as the mean ± SEM. The comparison between two groups was analysed using Student’s t-test. A value of *P* < 0.05 was considered statistically significant.

## Additional Information

**How to cite this article:** Wang, C. *et al*. MicroRNA-199a-5p promotes tumour growth by dual-targeting PIAS3 and p27 in human osteosarcoma. *Sci. Rep.* **7**, 41456; doi: 10.1038/srep41456 (2017).

**Publisher's note:** Springer Nature remains neutral with regard to jurisdictional claims in published maps and institutional affiliations.

## Supplementary Material

Supplementary Information
