# Supplementary material for: MicroRNA-199a-5p promotes tumour growth by dual-targeting PIAS3 and p27 in human osteosarcoma
Source: Sci Rep. 2017 Jan 25;7:41456. doi: 10.1038/srep41456 (PMC5264164; doi:10.1038/srep41456)
Supplement: Supplementary Information [file srep41456-s1.doc]

**MicroRNA-199a-5p promotes tumour growth by dual-targeting PIAS3 and p27 in human osteosarcoma**

**Chen Wang,^1^ Ximing Ba,^1^ Yu Guo,^1^ Defang Sun,^1^ HaoyangJiang,^1^Wentao Li,^1^**

**Zhen Huang,^1^ Guangxin Zhou,^3^ Sujia Wu,^3^ Junfeng Zhang,^1,2*^ Jiangning Chen^1,2*^**

^1^State Key Laboratory of Analytical Chemistry for Life Sciences and Collaborative Innovation Center of Chemistry for Life Sciences, State Key Laboratory of Pharmaceutical Biotechnology, School of Life Sciences, Nanjing University, Nanjing 210023, P. R. China

^2^Department of Orthopaedics, Jinling Hospital, School of Medicine, Nanjing University, Nanjing 210002, P. R. China

*Correspondence should be addressed to Jiangning Chen ([jnchen@nju.edu.cn](mailto:jnchen@nju.edu.cn)) or Junfeng Zhang (jfzhang@nju.edu.cn).

**Supplementary Table 1**

The information of osteosarcoma patients

|  | **Number(n)** | **Proportion(%)** |
| --- | --- | --- |
| ***All cases*** | 8 | 100 |
| ***Gender*** |  |  |
| Male | 5 | 62.5 |
| Female | 3 | 37.5 |
| ***Age(yr)*** |  |  |
| ≤ 15 | 3 | 37.5 |
| > 15 | 5 | 62.5 |
| ***Location*** |  |  |
| Femur | 5 | 62.5 |
| Tibia | 2 | 25 |
| Humerus | 1 | 12.5 |

**Supplementary Table 2**

The primers for qRT-PCR assay of miRNAs and genes

| **Primer Name** | **Primer Sequence (5’-3’)** |
| --- | --- |
| miR-199a-5p RT prime | CTCAACTGGTGTCGTGGAGTCGGCAATTCAGTTGAGTGGGGTAT |
| miR-199a-5p Reverse prime | TGGTGTCGTGGAGTCG |
| miR-199a-5p Forward prime | ACACTCCAGCTGGG TGTCAGTTTGTCAAAT |
| U6 RT prime | AACGCTTCACGAATTTGCGT |
| U6 Reverse prime | AACGCTTCACGAATTTGCGT |
| U6 Forward prime | CTCGCTTCGGCAGCACA |
| β-actin RT prime | random primer |
| β-actin Reverse prime | GACTGCTGTCACCTTCACCGTTC |
| β-actin Forward prime | GACTTAGTTGCGTTACACCCTTTCTTG |
| PIAS3 RT prime | random primer |
| PIAS3 Reverse prime | GCCTCACCAGGTACACAGAC |
| PIAS3 Forward prime | GACTCTCAGCCACTGTTCCC |
| P27 RT prime | random primer |
| P27 Reverse prime | CCTTATTCCTTCGCTGGACG |
| P27 Forward prime | AGGGCAAGTACGAGTGGCA |
| PCNA RT prime | random primer |
| PCNA Reverse prime | TCTTCATTGCCGGCGCATT |
| PCNA Forward prime | CAGGGCTCCATCCTCAAGAA |
| Ki67 RT prime | random primer |
| Ki67 Reverse prime | TCGGGCTGCCAGATAGAGTC |
| Ki67 Forward prime | ATGGATAAGCGCACGGATGAAT |

**Supplementary Table 3**

**Presumed miRNA-targets predicted by TargetScan (T), miRanda (M) and PicTar (P).**

| **Name** | **Gene ID** | | **Full Target Name** | **microRNA(algorithms)** |
| --- | --- | --- | --- | --- |
| PIAS3 | | 10401 | protein inhibitor of activated STAT3 | miR-199a-5p(M+P+T) |
| CDKN1B(p27) | | 1027 | cyclin-dependent kinase inhibitor 1B | miR-199a-5p(M+P+T) |
| RAB10 | | 10890 | member RAS oncogene family | miR-199a-5p(M+P+T) |
| DUSP14 | | 11072 | dual specificity phosphatase 14 | miR-199a-5p(M+P+T) |
| DBF4 | | 10926 | DBF4 zinc finger | miR-199a-5p(M+P+T) |
| RBPMS | | 11030 | RNA binding protein with multiple splicing | miR-199a-5p(M+P+T) |
| PDPN | | 10630 | podoplanin | miR-199a-5p(M+P+T) |
| CLTC | | 1213 | clathrin, heavy chain (Hc) | miR-199a-5p(M+P+T) |
| ATP9A | | 10079 | ATPase phospholipid transporting 9A (putative) | miR-199a-5p (M+P+T) |
| USPL1 | | 10208 | ubiquitin specific peptidase like 1 | miR-199a-5p (M+P+T) |
| SRRM1 | | 10250 | serine and arginine repetitive matrix 1 | miR-199a-5p (M+P+T) |
| HBS1L | | 10767 | HBS1 like translational GTPase | miR-199a-5p (M+P+T) |
| KLHL32 | | 114792 | kelch like family member 32 | miR-199a-5p (M+P+T) |
| TMEM132B | | 114795 | transmembrane protein 132B | miR-199a-5p (M+P+T) |
| LYSMD3 | | 116068 | LysM domain containing 3 | miR-199a-5p (M+P+T) |
| SNX20 | | 124460 | sorting nexin 20 | miR-199a-5p (M+P+T) |
| CCDC43 | | 124808 | coiled-coil domain containing 43 | miR-199a-5p (M+P+T) |
| ZNF440 | | 126070 | zinc finger protein 440 | miR-199a-5p (M+P+T) |
| COL19A1 | | 1310 | collagen type XIX alpha 1 chain | miR-199a-5p (M+P+T) |

**Supplementary Figures**


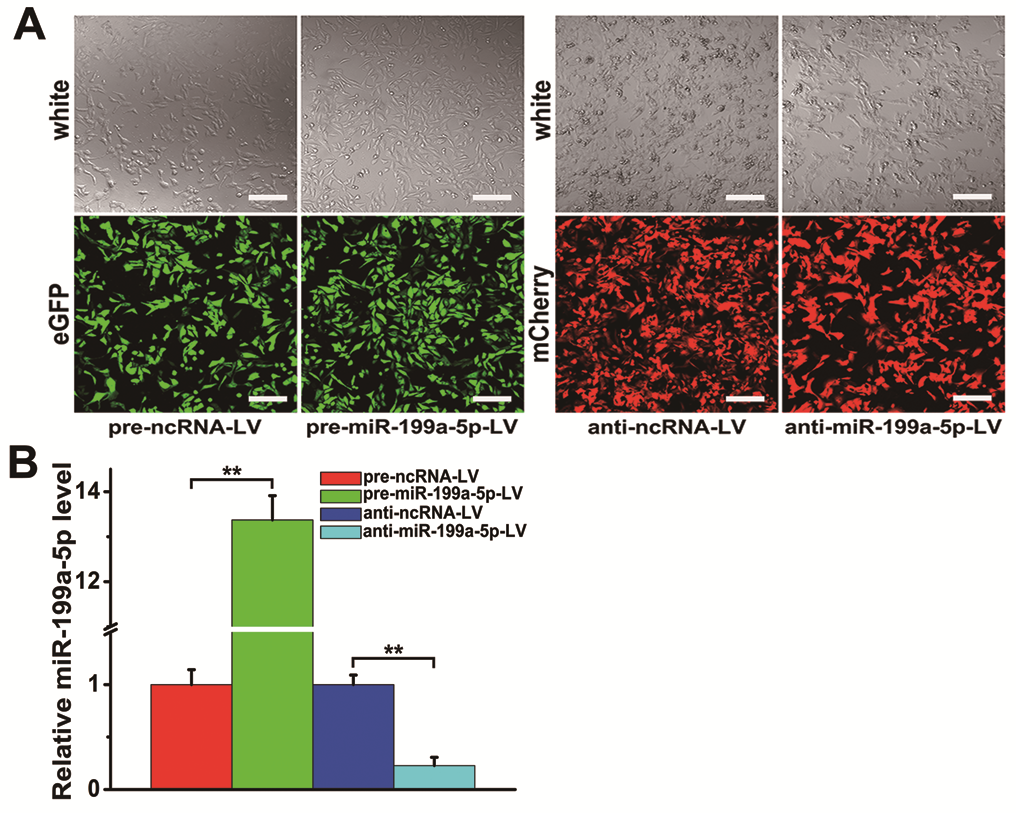


**Figure S1 The construction and identification of MNNG/HOS cells stably expressing/inhibiting miR-199a-5p.** (A) MiR-199a-5p stably expressing/inhibiting MNNG/HOS cells were sorted in the presence of 10 μg/ml puromycin (Sigma-Aldrich) since the cells have puromycin-resistance marker carried by the lentivirus. The pre/anti-miR-199a-5p-LV group represents the stably expressing/inhibiting miR-199a-5p cells and the pre/anti-ncRNA-LV group represents the control cells. The pictures of cells were captured on a Nikon confocal microscope based on the expression of GFP or mCherry. Pictures were imaged at ×200 magnification. Scale bar, 100 μm. (B) Relative miR-199a-5p level in MNNG/HOS cells stably expressing/inhibiting miR-199a-5p was confirmed using qRT-PCR analysis.


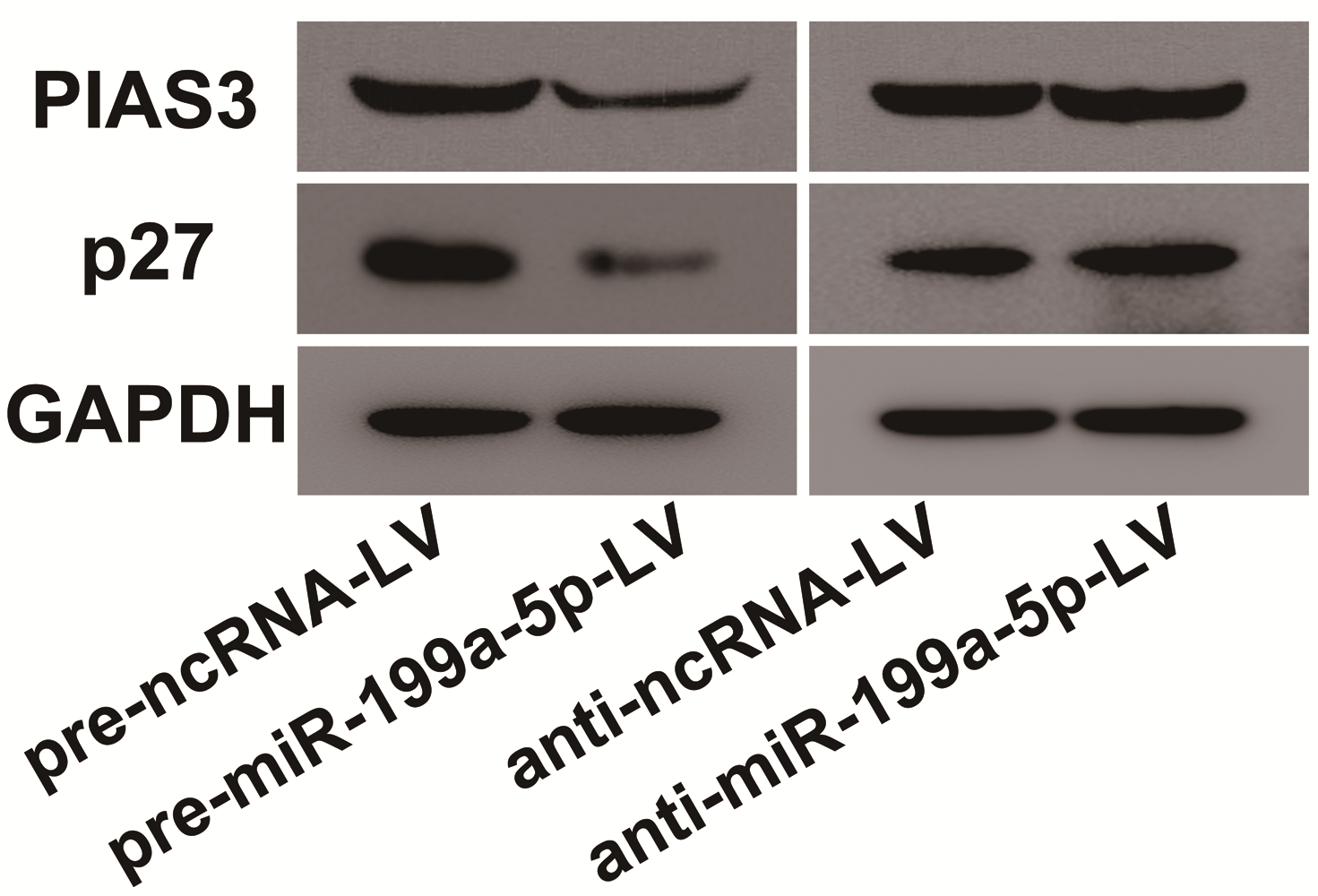


**Figure S2 The protein levels of PIAS3 and p27 in MNNG/HOS cells stably expressing/inhibiting miR-199a-5p were detected by western blotting.**

**
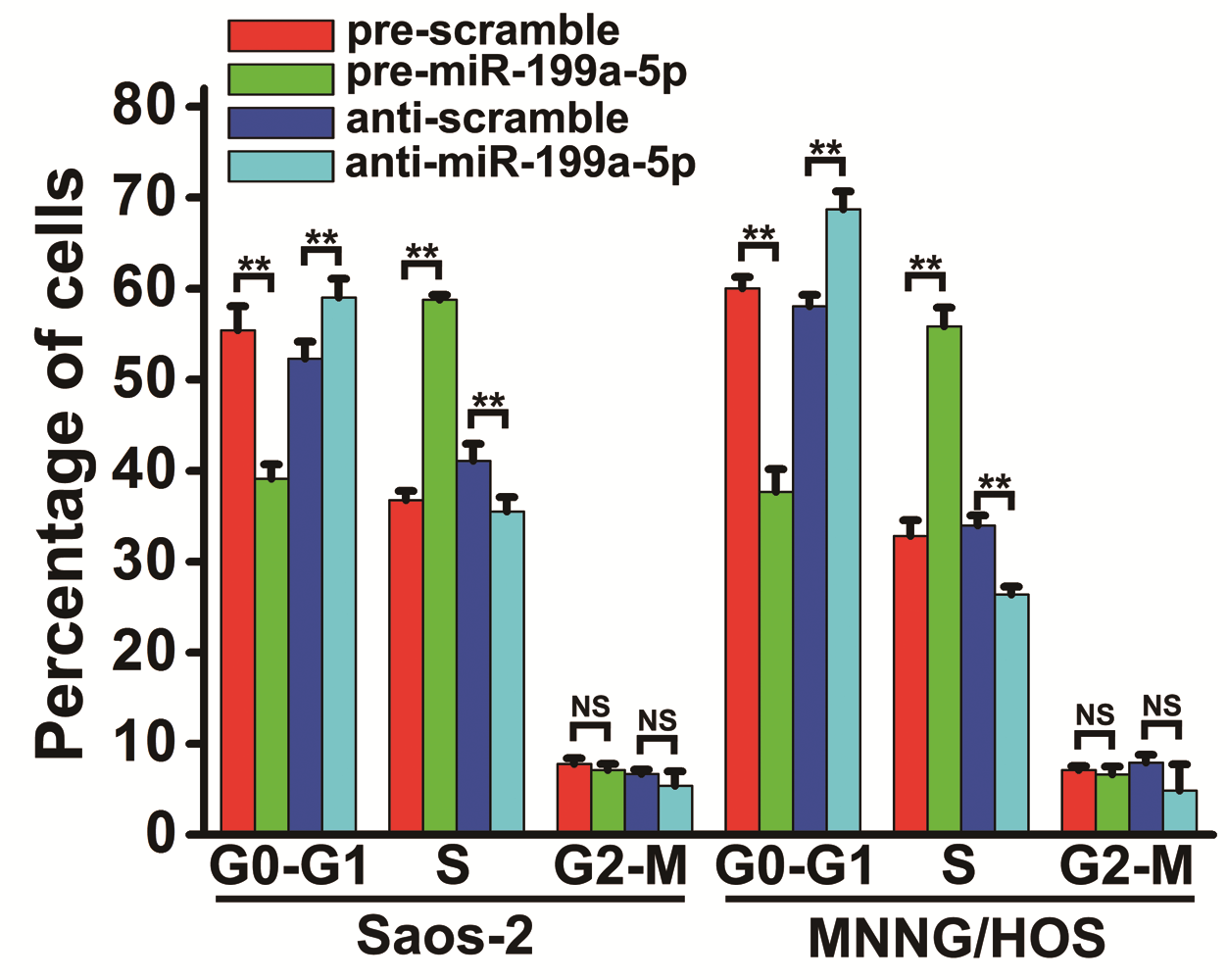
**

**Figure S3 Cell cycle analysis of Saos-2 and MNNG/HOS cells after transfected with pre/anti-miR-199a-5p or the corresponding control. The percentage of cells in G0-G1, S, G2-M phase is shown as histograms.**

**
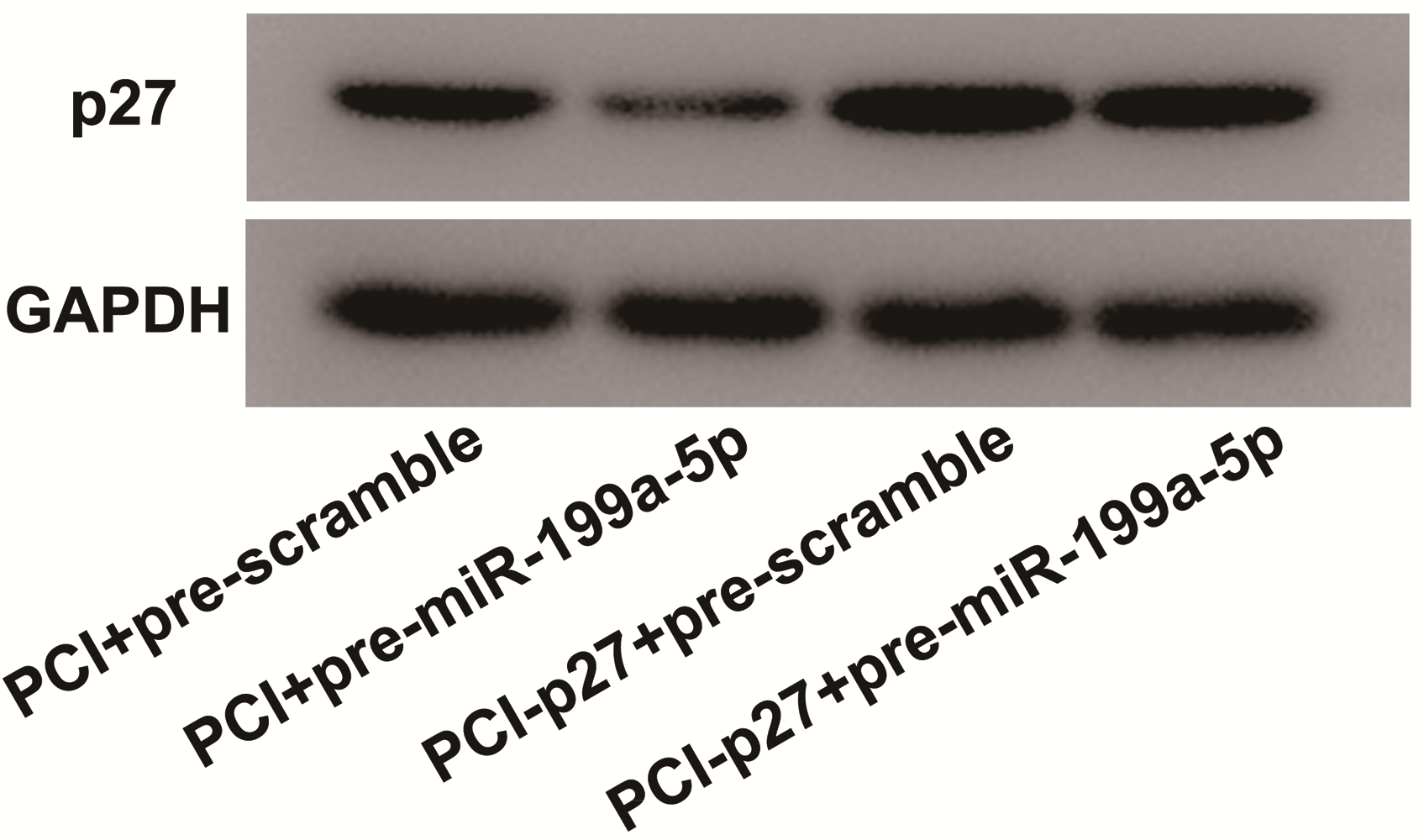
**

**Figure S4 The protein levels of p27 in MNNG/HOS cells** **after transfected with pre-scramble plus PCI plasmid, pre-miR-199a-5p plus PCI plasmid, pre-scramble plus PCI-p27 plasmid or pre-miR-199a-5p plus PCI-p27 plasmid for 48 hours.**

**
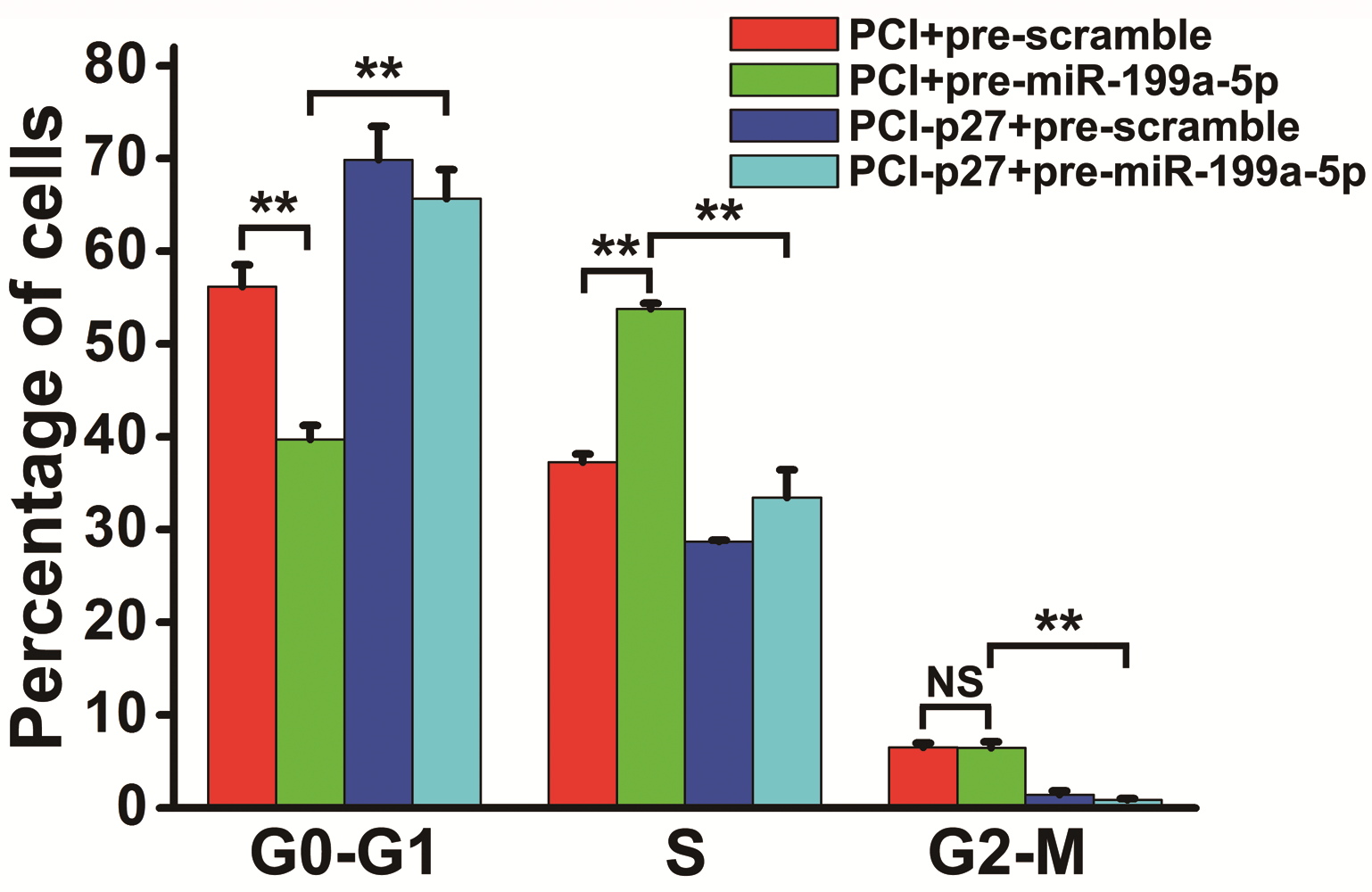
**

**Figure S5 Cell cycle analysis of MNNG/HOS cells after transfected with pre-scramble plus PCI plasmid, pre-miR-199a-5p plus PCI plasmid, pre-scramble plus PCI-p27 plasmid or pre-miR-199a-5p plus PCI-p27 plasmid for 48h. The percentage of cells in G0-G1, S, G2-M phase is shown as histograms.**
